# Activation status of mucosal-associated invariant T cells reflects disease activity and pathology of systemic lupus erythematosus

**DOI:** 10.1186/s13075-017-1257-5

**Published:** 2017-03-14

**Authors:** Asako Chiba, Naoto Tamura, Kazunori Yoshikiyo, Goh Murayama, Mie Kitagaichi, Ken Yamaji, Yoshinari Takasaki, Sachiko Miyake

**Affiliations:** 1 Department of Immunology, Juntendo University School of Medicine, 2-1-1 Hongo, Bunkyo-ku, Tokyo, 113-8421 Japan; 2 Department of Internal Medicine and Rheumatology, Juntendo University School of Medicine, Tokyo, Japan; 3 Asubio Pharma Co., Ltd., Kobe, Japan

**Keywords:** Systemic lupus erythematosus, Innate-like lymphocytes, Mucosal-associated invariant T cells, Activation marker, Disease activity index, Antigen presentation, Cytokines

## Abstract

**Background:**

Mucosal-associated invariant T (MAIT) cells are innate-like lymphocytes constituting a large proportion of peripheral blood T cells expressing αβ T-cell receptor in humans. In this study, we aimed to investigate their involvement in systemic lupus erythematosus (SLE).

**Methods:**

Peripheral blood MAIT cells from patients with SLE were assessed for their frequency, activation markers, and cell death by flow cytometry. The correlation between plasma cytokine levels and CD69 expression on MAIT cells was analyzed. The major histocompatibility complex class I-related protein MR1-restricted antigen-presenting capacity of antigen-presenting cells was investigated. Cytokine-mediated activation of MAIT cells in the absence of exogenous antigens was also examined.

**Results:**

The frequency of MAIT cells was markedly reduced in SLE. The reduced number of MAIT cells was not attributable to the downregulation of surface markers, but it was partially due to the enhanced cell death of MAIT cells, possibly by activation-induced cell death. The CD69 expression levels on MAIT cells in SLE correlated with disease activity. Moreover, monocytes from patients with SLE exhibited increased ability to induce MAIT cell activation. The plasma concentration of interleukin (IL)-6, IL-18, and interferon (IFN)-α positively correlated with the expression levels of CD69 on MAIT cells in SLE. MAIT cells were activated by cytokines, including IFN-α, IL-15, and IL-12 plus IL-18, in the absence of exogenous antigens.

**Conclusions:**

These results suggest that MAIT cells reflect the pathological condition of SLE and that their activated status correlates with presence of disease.

## Background

Mucosal-associated invariant T (MAIT) cells are a unique population of innate-like lymphocytes that is restricted by major histocompatibility complex class I-related protein (MR1) and expresses the semi-invariant T-cell receptor (TCR) Vα7.2-Jα33 in humans and Vα19-Jα33 in mice with a limited set of Vβ chains [1, 2]. Similarly to other innate-like lymphocytes, such as natural killer (NK) cells, γδT cells, and invariant natural killer T (iNKT) cells, MAIT cells rapidly exert effector functions upon activation without the need to undergo clonal expansion [3, 4]. MAIT cells were originally named after their preferential location at mucosal tissues, where they are involved in the first-line defense against exogenous stimuli [2]. Interestingly, MAIT cells are absent in the peripheral blood of germ-free mice [2]. MAIT cells have been shown to react to antigen-presenting cells in the presence of certain types of microbes in vitro and to protect mice against pathogenic bacteria in vivo [5–7]. Therefore, MAIT cells are thought to recognize microbial antigens, and Kjer-Nielsen et al. recently elucidated that bacteria-derived vitamin B_2_ metabolites bind to MR1 and activate MAIT cells [8].

Other recent studies revealed that MAIT cells are abundant in human peripheral blood and constitute between 1% and 10% of all αβT cells [4, 9]. They express high levels of CD161, an NK receptor also known as NKR-P1A, and exhibit effector memory phenotypes in peripheral blood, presumably owing to their prior exposure to microbial antigens from commensal bacteria. Similarly to other innate-like T cells such as γδT cells and iNKT cells, MAIT cells can be fully activated by cytokines in the absence of exogenous antigens [10–12]. Previously, we demonstrated that murine MAIT cells stimulated with interleukin (IL)-23 produce IL-17, whereas IL-1β induces MAIT cell proliferation [10]. Human MAIT cells are also activated by the combination of IL-12 and IL-18 and produce interferon (IFN)-γ. These activation mechanisms of MAIT cells indicate that they could be involved in various types of immune responses besides antimicrobial immunity. Indeed, whereas MAIT cells exacerbate joint inflammation in arthritis models, they suppress neuroinflammation in experimental autoimmune encephalomyelitis and colitis in a trinitrobenzenesulfonic acid-induced colitis model [10, 13, 14].

Systemic lupus erythematosus (SLE) is an autoimmune disease characterized by the production of autoantibodies. The activation of autoreactive B and T cells is a hallmark of lupus pathology, although other features include numerical and functional abnormalities of innate immune cells and innate-like lymphocytes. In SLE, the frequencies of NK cells, iNKT cells, and γδT cells are decreased, and the killing activity of NK cells and the cytokine production of iNKT cells are reduced [15–21]. In addition, the proliferative capacity of lupus γδT cells is diminished [21]. Although it is not known whether these abnormalities are a cause or consequence of the pathological process underlying SLE, the activated status and functional abnormalities are clearly associated with the pathology.

Recently, Cho et al. reported that levels of MAIT cells were reduced in patients with SLE [22]. MAIT cell deficiency is associated with these cells’ poor responsiveness upon activation and elevated programmed cell death protein 1 (PD-1) expression. Numerical and functional deficiencies of iNKT cells may also contribute to the malfunction of MAIT cells. We also demonstrate that the frequency of MAIT cells was markedly reduced in patients with SLE. However, contrary to the findings of Cho et al., we show that MAIT cells in SLE are greatly activated and that their activation is correlated with disease activity. The reduction of MAIT cell frequency was not due to a downregulation of surface markers, but rather was partially caused by activation-induced cell death. We elucidate two possible mechanisms of MAIT cell activation in SLE. First, we demonstrate that monocytes in patients with SLE have an increased capacity to present MR1 antigens and activate MAIT cells. Second, we show that the plasma levels of IL-6, IL-18, and IFN-α positively correlate with the activated status of MAIT cells in SLE, and that MAIT cells are activated by cytokines, including IL-18 and IFN-α, in the absence of exogenous antigens, indicating that inflammatory cytokines may also be responsible for the activation of MAIT cells in lupus. Taken together, MAIT cells reflect the pathological condition of SLE, and their activated status correlates with presence of disease. These findings suggest that MAIT cells may be associated with the pathogenesis of SLE.

## Methods

### Human samples

We obtained peripheral blood from a total of 53 patients with SLE (49 females and 4 males, median age 36.0 years [IQR 30.0–43.0], median disease duration 6.0 years [IQR 1.0–11.0]) diagnosed according to the American College of Rheumatology revised criteria for SLE [23] and 48 healthy control subjects (HCs) after gaining informed consent in accordance with local ethics committee guidelines of Juntendo University. HCs were matched to patients with SLE for sex and age. The characteristics of patients with SLE and HCs are shown in Table 1. Disease activity was measured using the Systemic Lupus Erythematosus Disease Activity Index (SLEDAI) [24], and a SLEDAI score ≥5 was defined as active disease. This study was conducted with the approval of the regional ethics committee at Juntendo University Hospital.

**Table 1:** Characteristics of healthy control subjects and patients with lupus

	HCs	Patients with SLE
Number	48	53
Females/males, *n*	43/5	49/4
Age, years	37.5 (28.3–43.8)	36.0 (30.0–43.0)
Disease duration, years		6.0 (1.0–11.0)
SLEDAI score		6.0 (2.0–15.5)
SLEDAI <5, *n*		25
SLEDAI ≥5, *n*		28
Medications		
Medication-naïve, *n*		7
Prednisone, *n*		46
Prednisone dose, mean ± SD (mg/day)		11.0 (5.0–22.5)
Immunosuppressive agent,^a^ *n*		13

*Abbreviations: HCs* Healthy control subjects, *SLE* Systemic lupus erythematosus, *SLEDAI* Systemic Lupus Erythematosus Disease Activity Index

Values are *n* or median (interquartile range)

^a^Azathioprine, cyclosporine, cyclophosphamide, mizoribine, mycophenolate mofetil, tacrolimus

### Flow cytometric analysis

Peripheral blood mononuclear cells (PBMCs) were purified from heparinized blood by centrifugation over a Ficoll-Paque gradient (GE Healthcare, Uppsala, Sweden). PBMCs were incubated with Fc receptor blocking reagent (Miltenyi Biotec, Bergisch Gladbach, Germany), and cell surface staining was performed using the following monoclonal antibodies (mAbs) and tetramers: TCR pan-γ/δ-fluorescein isothiocyanate (FITC), CD8β-phycoerythrin (PE)-Texas Red electron-coupled dye (Beckman Coulter, Indianapolis, IN, USA); CD3-allophycocyanin (APC)-H7, CD4-APC-H7, CD8a-APC-H7, CD3-AmCyan, CD3-BD Horizon V500 (BD Biosciences, San Jose, CA, USA); TCR Vα7.2-PE, TCR Vα7.2-APC, TCRγ/δ-APC, CD3-peridinin chlorophyll (PerCP)/cyanine 5.5 (Cy5.5), CD161-PerCP/Cy5.5, CD161-Brilliant Violet 421, CD4-Alexa Fluor 700, CD69-Alexa Fluor 700, CD69-Brilliant Violet 605, CD95-Brilliant Violet 421, (BioLegend, San Diego, CA, USA); CD161-APC (eBioscience, San Diego, CA, USA); and CD1d/PBS-57 tetramer-APC (National Institutes of Health Tetramer Core Facility, Atlanta, GA, USA). Staining with 7-aminoactinomycin D (7-AAD; BD Biosciences) was performed to discern dead cells. In most experiments, samples were fixed using BD stabilizing fixative (BD Biosciences). Intracellular staining of active caspase-3 was performed using the caspase-3 active form mAb apoptosis kit with FITC (BD Biosciences). Data were acquired by fluorescence-activated cell sorting (FACS) on an LSRFORTESSA (BD Biosciences), and the percentages of each cell population and mean fluorescence intensities (MFIs) were analyzed with FlowJo software (FlowJo LLC, Ashland, OR, USA).

### Single-cell polymerase chain reaction

Cells were single-cell-sorted using the BD FACSAria II system (BD Biosciences) into catch buffer (10 mM Tris, pH 8.0) containing 40 U/μl of RNasin ribonuclease inhibitor (Promega, Madison, WI, USA). Reverse transcriptase-polymerase chain reaction (RT-PCR) was performed using a four-primer mixture and the QIAGEN OneStep RT-PCR Kit (QIAGEN, Germantown, MD, USA) following the manufacturer’s instructions. The following primers were used: AV7.2-AJ33-S, CTGGATGGTTTGGAGGAGAA; AV7.2-AJ33-AS, GCGCCCCAGATTAACTGATA; TRAC-A, TGCCTATTCACCGATTTTGA; and TRAC-AS, GCAGCGTCATGAGCAGATTA.

### Cell culture

PBMCs were cultured in 96-well flat-bottom plates in RPMI 1640 medium (Thermo Fisher Scientific, Waltham, MA, USA) supplemented with 10% FBS, 2 mM l-glutamine, 50 U/ml penicillin, and 50 μg/ml streptomycin (all from Thermo Fisher Scientific). PBMCs were stimulated with either immobilized anti-CD3 mAb (OKT3, 1 μg/ml; American Type Culture Collection, Manassas, VA, USA) or IL-6, IL-12p70 (PeproTech, Rocky Hill, NJ, USA), IL-13, IL-15 (BioLegend), IL-18 (Medical & Biological Laboratories Co. [MBL], Nagoya, Japan), granulocyte-macrophage colony-stimulating factor (GM-CSF), IFN-α (R&D Systems, Minneapolis, MN, USA), or a major histocompatibility complex class I-related protein ligand (MR1L; 10 μM). The cytokine concentrations for stimulation were 50 ng/ml, except for IFN-α (100 U/ml). 7-Methyl-8-d-ribityllumazine was synthesized following previously reported procedures [25] and used as an MR1L.

### Proliferation assay

PBMCs were labeled using the CellTrace Violet Cell Proliferation Kit (Thermo Fisher Scientific) and cultured in 96-well flat-bottom plates coated with anti-CD3 mAb (1 μg/ml) and anti-CD28 mAb (CD28.2, 1 μg/ml; BioLegend). Seven days later, cells were stained with surface markers and 7-AAD, and CellTrace low-dividing cells or 7-AAD-positive dead cells were analyzed by FACS.

### Activation of human MAIT cells by MR1L

B cells or monocytes were sorted from PBMCs using anti-CD19 or anti-CD14 microbeads (Miltenyi Biotec), respectively, following the manufacturer’s instructions. MAIT cells were sorted from PBMCs of HCs using the BD FACSAria II system (BD Biosciences) or MoFlo Astrios cell sorter (Beckman Coulter) as described elsewhere [26]. B cells or monocytes (1 × 10^4^ cells) were cultured for 18 h with MAIT cells (5 × 10^4^ cells) in the presence of MR1L (10 μM).

### Cytokine measurement

Cytokine levels were measured using sandwich enzyme-linked immunosorbent assays for IL-18 (MBL) and IFN-α (PBL Assay Science, Piscataway, NJ, USA) or a Bio-Plex assay (Bio-Rad Laboratories, Hercules, CA, USA) for other cytokines.

### Statistical analysis

All data were analyzed using Prism 6 software (GraphPad Software Inc., La Jolla, CA, USA), and differences between the groups were analyzed using the Mann–Whitney *U* test, the Kruskal-Wallis test followed by Dunn’s multiple comparisons test, or the Wilcoxon rank-sum test. The significance level was set at *p* < 0.05. Associations between two variables were analyzed using Spearman’s correlation.

## Results

### The frequency of MAIT cells is reduced in peripheral blood of patients with SLE

**Fig. 1:**
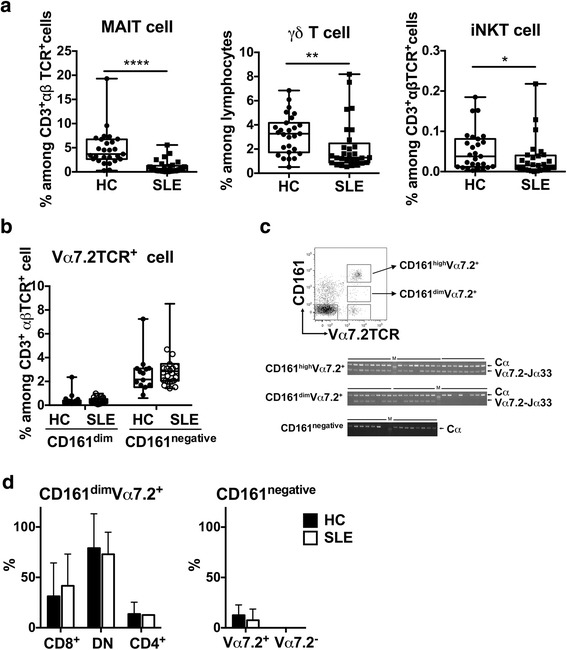
Decreased frequency of mucosal-associated invariant T (MAIT) cells in patients with systemic lupus erythematosus (SLE). **a**, **b** Peripheral blood mononuclear cells (PBMCs) were stained with monoclonal antibodies against CD3, T-cell receptor (TCR) γδ, Vα7.2, CD161, and CD1d/PBS-57 tetramer, and analyzed by flow cytometry. **a** Percentages of MAIT cells (Vα7.2^+^CD161^high^CD3^+^TCRγδ^−^cells), γδT cells (CD3^+^TCRγδ^+^), and invariant natural killer T (iNKT) cells (CD3^+^TCRγδ^−^CD1d/PBS-57 tetramer^+^) are shown. **b** Percentages of CD161^dim^ and CD161^negative^ Vα7.2 TCR^+^ cells among αβT cells. Each symbol represents the value of one individual. The box plot indicates the first and third quartiles, and the *middle line* indicates the median. Whiskers indicate the minimum and maximum. * *p* < 0.05, ** *p* < 0.01, **** *p* < 0.0001 (Mann–Whitney *U* test). **c**, **d** Invariant Vα7.2-Jα33 TCR use in Vα7.2 TCR^+^ T cells. **c** Representative fluorescence-activated cell sorting staining profile and gating strategy for single-cell sorting of T-cell populations, including MAIT cells. Single-cell amplification of TCR α constant (Cα) and AV7.2-AJ33 was performed. A representative agarose gel electrophoresis image showing TCR segments containing Cα and AV7.2-AJ33 in each T-cell subpopulation from a healthy control (HC) are shown. **d** Summary of AV7-AJ33-bearing cells in each T-cell subpopulation determined by single-cell polymerase chain reaction

First, by FACS analysis, we investigated the frequencies of MAIT cells and other innate-like T cells, including iNKT cells and γδT cells, in peripheral blood from patients with SLE. As previously reported, the proportions of all innate-like lymphocytes examined in the study were reduced in patients with SLE (Fig. 1a). The frequency of MAIT cells was markedly reduced in patients with SLE (median 0.65%, IQR 0.20–1.28) compared with that in HCs (median 3.69%, IQR 2.65–6.73) (Fig. 1a). In contrast, the reduction of other innate-like lymphocyte subsets was milder than that of MAIT cells (Fig. 1a). MAIT cells can be distinguished by flow cytometry as Vα7.2 TCR^+^ αβT cells with a high expression of CD161. Because lymphocytes may downregulate surface molecules, we investigated whether the reduction of MAIT cell frequency in SLE was due to the downregulation of the surface markers. There was no increase of CD161^dim^ or CD161^negative^ Vα7.2 TCR^+^ cells in patients with SLE compared with HCs (Fig. 1b). Next, we performed single-cell PCR analysis for the expression of Vα7.2-Jα33 TCR to evaluate whether αβT cells expressing invariant Vα7.2-Jα33 TCR were contaminated among other αβT cell populations in SLE. As shown in Fig. 1d, the proportions of invariant Vα7.2-Jα33 expression in CD161^dim^ and CD161^negative^ cells were not increased in patients with SLE compared with HCs. These results indicate that the decreased frequency of MAIT cells in patients with SLE did not result from a downregulation of surface markers.

### Cell death of MAIT cells is enhanced in SLE

**Fig. 2:**
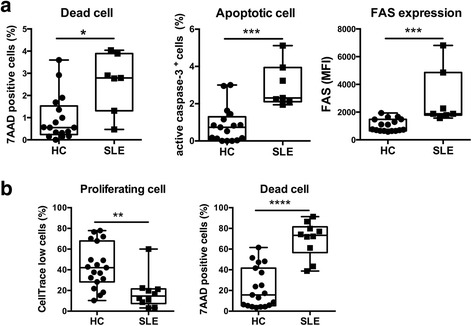
Enhanced cell death of lupus mucosal-associated invariant T (MAIT) cells. **a** Freshly isolated peripheral blood mononuclear cells (PBMCs) from patients with systemic lupus erythematosus (SLE) and healthy control subjects (HCs) were stained with monoclonal antibodies (mAbs) against CD3, T-cell receptor (TCR) γδ, Vα7.2, CD161, and CD95 (FAS). 7-Aminoactinomycin D (7-AAD) staining, intracellular active caspase-3 staining, and surface FAS expression of MAIT cells were analyzed by fluorescence-activated cell sorting (FACS). **b** PBMCs were labeled with CellTrace Violet and cultured in plates coated with anti-CD3 mAb (1 μg/ml) and anti-CD28 mAb (1 μg/ml). After 7 days, cell proliferation and 7-AAD-positive dead cells were analyzed by FACS. Each symbol represents the value of one individual. The box plot indicates the first and third quartiles, and the *middle line* indicates the median. Whiskers indicate the minimum and maximum. * *p* < 0.05, ** *p* < 0.01, *** *p* < 0.001, **** *p* < 0.0001 (Mann–Whitney *U* test). *MFI* Mean fluorescence intensity

Next, we assessed whether the reduced frequency of MAIT cells was due to increased cell death. As expected, the levels of both 7-AAD- and active caspase-3-positive cells were increased in MAIT cells in patients with SLE (median 2.79 [IQR 1.31–3.89], 2.31 [IQR 2.11–3.94], respectively) (Fig. 2a) compared with the HCs (median 0.55 [IQR 0.24–1.53]; 0.73 [0.08–1.30], respectively) (Fig. 2a), indicating that cell death is enhanced in MAIT cells in lupus. Because FAS expression is increased on MAIT cells in SLE (median MFI for HCs 756 AU [IQR 647–1474] vs median MFI for SLE 1879 AU [1786–4855]) (Fig. 2a), we speculate that apoptosis occurs in MAIT cells through activation-induced cell death. Notably, MAIT cells from patients with lupus were prone to death upon in vitro stimulation. The percentages of dividing cells among activated MAIT cells from patients with SLE (median 14.60% [IQR 7.40–21.50]) were lower than those of cells from HCs (median 42.00% [IQR 28.30–67.90]), as shown in Fig. 2b. Instead, more MAIT cells were positive for 7-AAD after culture in SLE (median SLE 73.25 [IQR 56.65–81.50] vs median HCs 15.70 [IQR 5.30–41.60]).

### MAIT cells are activated in SLE, which reflects disease activity

**Fig. 3:**
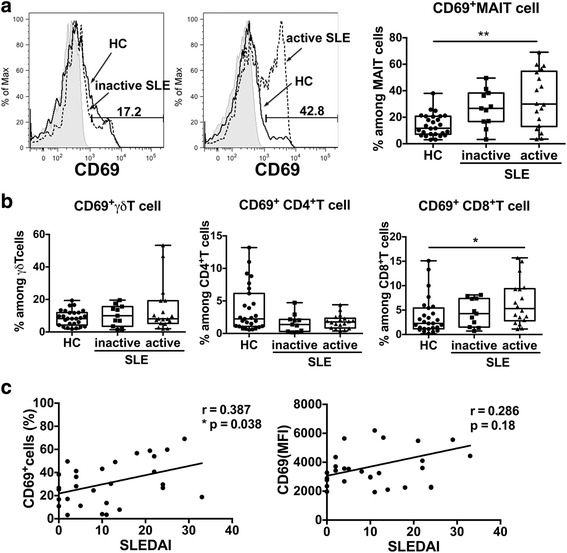
Mucosal-associated invariant T (MAIT) cell activation and correlation of CD69^+^ MAIT cell frequency with disease activity in systemic lupus erythematosus (SLE). CD69 expression on MAIT cells (**a**) and other T-cell subsets (**b**), including γδT cells, CD4^+^ T cells, and CD8^+^ T cells, were analyzed by flow cytometry. **a** Representative examples of CD69 expression on MAIT cells and the percentages of CD69^+^ cells among each MAIT cell population in patients with SLE are shown. The *gray histogram* indicates isotype control. The relative levels of CD69^+^ MAIT cells are increased in patients with SLE with active disease compared with healthy control subjects (HCs). *p < 0.05, ** *p* < 0.01 (Kruskal-Wallis test followed by Dunn’s multiple comparisons test). Each symbol represents the value of one individual. The box plot indicates the first and third quartiles, and the *middle line* indicates the median. Whiskers indicate the minimum and maximum. **c** Correlations between Systemic Lupus Erythematosus Disease Activity Index (SLEDAI) score and CD69^+^ MAIT cells or CD69 expression on MAIT cells. Symbols represent individual subjects. There was a positive correlation between the percentages of CD69^+^ cells among MAIT cells and the SLEDAI score. Correlations were analyzed using Spearman’s correlation analysis. *MFI* Mean fluorescence intensity

We next investigated the expression of CD69, an early activation marker on MAIT cells, to verify whether MAIT cells are activated in SLE. The percentages of CD69^+^ cells among MAIT cells tended to be higher in patients with SLE with inactive disease (median 26.70% [IQR 16.80–38.30]) than in healthy subjects (median 11.70% [IQR 7.22–20.80]), although there was no statistical difference between the two groups (Fig. 3a). In contrast, MAIT cells from patients with active disease showed an increased expression of CD69 (median 29.95% [IQR 13.00–54.68]) compared with MAIT cells in HCs. Interestingly, there was a positive correlation between the SLEDAI score and the percentage of CD69^+^ MAIT cells (Fig. 3c). There was no increased surface expression of CD69 on other T-cell subsets in patients with SLE compared with HCs (Fig. 3b). These results indicate that only the activation status of MAIT cells was associated with SLE disease activity.

### Monocytes from patients with SLE exert a profound effect on MAIT cell activation capacity

**Fig. 4:**
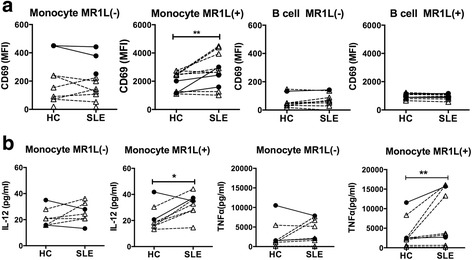
Augmented mucosal-associated invariant T (MAIT) cell-activating capacity of monocytes from patients with systemic lupus erythematosus (SLE). **a** Monocytes or B cells were sorted from peripheral blood mononuclear cells (PBMCs) and cocultured with allogenic MAIT cells in the absence or presence of major histocompatibility complex class I-related protein ligand (MR1L; 10 μM). After 18 h, the surface expression of CD69 on MAIT cells was analyzed by flow cytometry (**a**), and the cytokine levels in the culture supernatants were measured by enzyme-linked immunosorbent assay or Bio-Plex assay (**b**). Each symbol represents the mean fluorescence intensity (MFI) of CD69 on cells (**a**) or the concentration of interleukin (IL)-12 and tumor necrosis factor (TNF)-α (**b**) from cultures with antigen-presenting cells from healthy control subjects (HCs), patients with SLE with inactive disease (*closed circles*), or active disease (*open triangles*). Data from the same experiment are connected by *lines*. Statistical analysis was performed using the Wilcoxon matched-pairs signed-rank test, * *p* < 0.05, ** *p* < 0.01

To reveal the mechanisms of MAIT cell activation in SLE, we tested MAIT cell activation by antigen-presenting cells from patients with SLE. Monocytes or B cells were cocultured with human MAIT cells with MR1L for 18 h, and the surface expression of CD69 on MAIT cells was analyzed by flow cytometry. The MFIs of CD69 on MAIT cells cocultured with B cells from patients with SLE or from HCs were comparable. MAIT cells cultured with monocytes from patients with SLE in the presence of MR1L displayed higher levels of CD69 than did cells cultured with monocytes from HCs (Fig. 4a). The enhanced antigen-presenting capacity was observed for monocytes from patients with both active and inactive disease. Coculture with B cells or monocytes without MR1L did not activate MAIT cells.

To elucidate the mechanism by which monocytes from patients with SLE exerted this increased MAIT cell-activating capacity, we next measured concentrations of 19 cytokines (IL-1β, IL-2, IL-4, IL-5, IL-6, IL-7, IL-8, IL-10, IL-12, IL-13, IL-15, IL-17, IL-18, IL-23, GM-CSF, granulocyte colony-stimulating factor, IFN-α, IFN-γ, and tumor necrosis factor [TNF]-α), including those known to activate MAIT cells. The concentrations of IL-12 and TNF-α were increased in the culture with MR1L and monocytes from patients with SLE (Fig. 4b). Other cytokines, such as IL-15 or IL-18, were either undetectable or comparable between cultures with monocytes from either patients with SLE or HCs (data not shown). These results suggest that the enhanced MAIT cell-activating capacity of monocytes from patients with SLE was associated with their augmented IL-12 production.

### Cytokine-mediated activation of MAIT cells may occur in active SLE

**Fig. 5:**
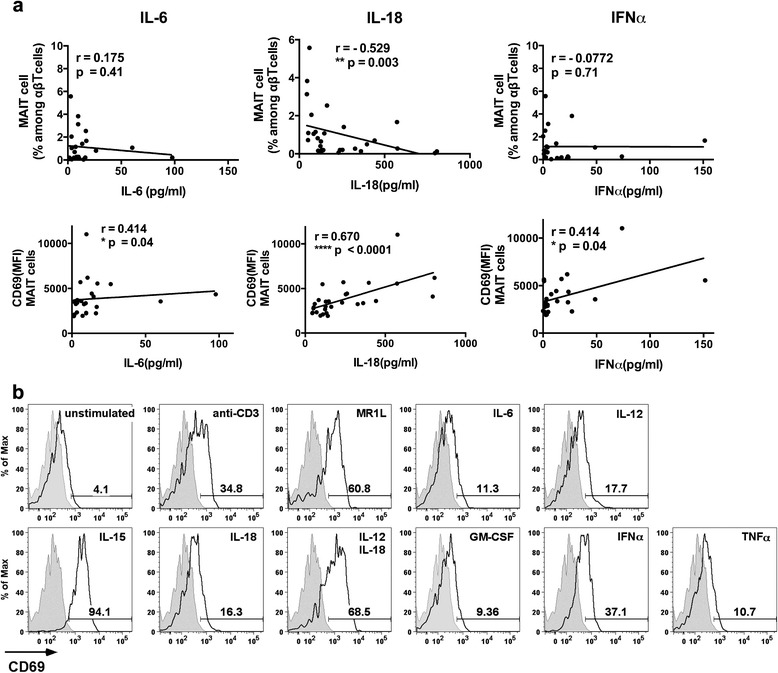
Cytokine-mediated activation of mucosal-associated invariant T (MAIT) cells. **a** Correlation between the plasma levels of cytokines with the percentage of MAIT cells or CD69 expression on MAIT cells was examined using Spearman’s correlation. Each symbol represents the value of one individual. **b** MAIT cell activation upon cytokine stimulation was analyzed. Peripheral blood mononuclear cells (PBMCs) from healthy volunteers were cultured in plates coated with anti-CD3 monoclonal antibody or in the presence of major histocompatibility complex class I-related protein ligand or cytokines, as indicated. After 18 h, CD69 expression was analyzed on MAIT cells stimulated or unstimulated. *Closed histograms* indicate isotype control. Values in the graphs indicate the percentages of CD69^+^ cells among stimulated MAIT cells. Representative data from three independent experiments are shown. *GM-CSF* Granulocyte-macrophage colony-stimulating factor, *IFN* Interferon, *IL* Interleukin, *MFI* Mean fluorescence intensity, *TNF* Tumor necrosis factor

CD69 expression on MAIT cells was positively correlated with disease activity. However, monocytes from patients with active and stable disease equally activated MAIT cells (Fig. 4a). Therefore, we assumed that there were other factors responsible for CD69 upregulation on MAIT cells in patients with active SLE. Human MAIT cells and γδT cells are known to be activated by cytokines such as IL-12 and IL-18. We hypothesized that the activation of these cells may be due to cytokines that are increased in patients with active lupus. As shown in Fig. 5a, the plasma level of IL-18 negatively correlated with the percentage of MAIT cells in SLE. We also found that the plasma concentrations of IL-6, IL-18, and IFN-α positively correlated with the CD69 expression on MAIT cells.

Next, we tested which cytokines that were elevated in plasma from patients with SLE could activate MAIT cells upon in vitro stimulation. As previously reported, in vitro stimulation with IL-15 and IL-12 plus IL-18 induced CD69 upregulation on MAIT cells (Fig. 5b). MAIT cells were also strongly activated by IFN-α, an important factor in lupus pathogenesis. In contrast, cytokines such as IL-6 had little effect on MAIT cells (Fig. 5b).

## Discussion

In the present study, we demonstrated that MAIT cell levels are decreased in the peripheral blood of patients with SLE, and that the reduction of MAIT cells in the periphery was due, at least in part, to activation-induced cell death. MAIT cells in SLE are activated, and the expression of the activation marker CD69 on these cells clearly correlated with disease activity. We revealed that MAIT cell activation was associated with an increased activation capacity of monocytes from patients with lupus. We also demonstrated that the elevated levels of certain inflammatory cytokines correlated with the activated state of MAIT cells in SLE, and that this may elicit the cytokine-mediated activation of MAIT cells. Taken together, our data reveal that MAIT cells reflect the pathogenesis of SLE and that their activated status strongly correlates with presence of disease.

Poor responses of iNKT cells and γδT cells upon stimulation have been reported in SLE, and the low responsiveness of iNKT was shown to be due to cell-intrinsic impairment in these cells, but not antigen-presenting cells [17, 18, 20, 21]. Authors of a previous report on lupus MAIT cells showed that the elevated PD-1 expression was accompanied by impaired IFN-γ production by these cells [22]. In our study, MAIT cells from patients with SLE also proliferated poorly upon activation, and this may also be due to PD-1 expression on lupus MAIT cells. However, because they expressed high levels of CD69 and FAS, we speculated that MAIT cells were already activated in vivo and were not able to respond to further stimulation in vitro. We revealed that monocytes exhibited an enhanced MR1-restricted antigen-presenting capacity in patients with SLE. As MR1-restricted antigens are vitamin B metabolites originating from biosynthetic pathways present in various types of microbes, including indigenous bacteria, lupus monocytes may be exposed to such antigens and subsequently induce activation of MAIT cells.

The CD69 expression on MAIT cells was markedly upregulated in patients with active disease, but there was a trend of increased CD69 expression on MAIT cells even in patients with inactive disease. This suggests that chronic activation of MAIT cells may underlie the pathogenesis of SLE. Monocytes exhibit an enhanced antigen-presenting capacity, in both patients with active disease and those with inactive disease, and this may be responsible for the chronic activation of MAIT cells in SLE. CD69 expression on MAIT cells in patients with lupus positively correlated with plasma concentrations of IL-6, IL-18, and IFN-α. Thus, once robust inflammation occurs, MAIT cells are fully activated by cytokines, which are highly expressed in active disease, and this in turn causes CD69 upregulation on MAIT cells.

MAIT cell deficiency in the peripheral blood could be related to the use of immunosuppressive agents, including corticosteroids [26]. Cho et al. showed that there was no correlation between MAIT cell frequencies and use of steroids or immunosuppressive drugs in patients with SLE. In this study, we also did not observe a correlation between the dose of corticosteroid and the percentage of MAIT cells or CD69 expression on MAIT cells. Reduced frequency of MAIT cells in peripheral blood has previously been demonstrated in patients with other autoimmune diseases, inflammatory disorders, and infectious diseases [5, 22, 27–33]. In many cases, the reduction of peripheral blood MAIT cells appears to be due to their migration into the inflamed tissues because MAIT cells have a propensity to migrate to the site of inflammation. Notably, MAIT cells constitutively express high levels of chemokine receptors such as C-C chemokine receptors CCR5 and CCR6 [9, 27]. IL-18-stimulated MAIT cells upregulate the surface expression of very late antigen-4, an integrin that mediates T-cell migration through its interaction with vascular cell adhesion molecule-1 (VCAM-1) [12]. Indeed, MAIT cells accumulate in lesions of patients with multiple sclerosis, rheumatoid arthritis, inflammatory bowel diseases, and mycobacterial infection [5, 12, 22, 28, 30, 34], and the activated status of MAIT cells positively correlated with both the plasma levels of IL-18 and disease activity in ulcerative colitis [28]. The reduction of MAIT cell frequency was related to the elevated serum concentration of IL-18 in multiple sclerosis [12]. Urinary levels of IL-18 and VCAM-1 were reported to be increased and associated with pathological events in lupus nephritis [35, 36], so MAIT cells may also migrate into inflamed tissues in SLE.

Previously, we demonstrated that human MAIT cells exerted suppressive activity against IFN-γ production by other T cells [27]. However, under inflammatory conditions, MAIT cells produced more proinflammatory cytokines than anti-inflammatory cytokines. Human adipose tissue MAIT cells produced IL-10, but in obese patients, they produced little IL-10 and more IL-17 [37, 38]. MAIT cells produce higher levels of IL-17 in patients with inflammatory diseases, including ulcerative colitis, ankylosing spondylitis, diabetes, and obesity [28, 29, 36–38]. Thus, it is possible that MAIT cells recruited to inflamed sites contribute to the organ damage in SLE. Further investigations using animal models are in progress to elucidate the role of MAIT cells in the pathogenesis of lupus.

## Conclusions

This study indicates that MAIT cells are affected by inflammatory conditions in SLE and that their activated status reflects disease activity. These findings suggest the possible involvement of MAIT cells in the pathogenesis of SLE.

## Abbreviations

7-AAD: 7-Aminoactinomycin D
APC: Allophycocyanin
CCR: C-C chemokine receptor
Cy5.5: Cyanine 5.5
FACS: Fluorescence-activated cell sorting
FITC: Fluorescein isothiocyanate
GM-CSF: Granulocyte-macrophage colony-stimulating factor
HC: Healthy control subject
IFN-α: Interferon-α
IL: Interleukin
iNKT: Invariant natural killer T cells
mAb: Monoclonal antibody
MAIT: Mucosal-associated invariant T cells
MFI: Mean fluorescence intensity
MR1: Major histocompatibility complex class I-related protein
MR1L: Major histocompatibility complex class I-related protein ligand
NK: Natural killer cells
PBMC: Peripheral blood mononuclear cell
PD-1: Programmed cell death protein 1
PE: Phycoerythrin
PerCP: Peridinin chlorophyll
RT-PCR: Reverse transcriptase-polymerase chain reaction
SLE: Systemic lupus erythematosus
SLEDAI: Systemic Lupus Erythematosus Disease Activity Index
TCR: T-cell receptor
TNF-α: Tumor necrosis factor-α
VCAM-1: Vascular cell adhesion molecule-1
